# Diabetic retinopathy severity detection using an improved Whale optimization algorithm and convolutional Kolmogorov-Arnold network

**DOI:** 10.3389/fmed.2026.1709872

**Published:** 2026-03-13

**Authors:** Ashit Kumar Dutta, Nasser Ali Aljarallah, Abdul Rahaman Wahab Sait

**Affiliations:** 1 Department of Computer Science and Information Systems, College of Applied Sciences, AlMaarefa University, Riyadh, Saudi Arabia; 2 King Salman Center for Disability Research, Riyadh, Saudi Arabia; 3 Department of Documents and Archive, Center of Documents and Administrative Communication, King Faisal University, Al Hofuf, Al-Ahsa, Saudi Arabia

**Keywords:** diabetic retinopathy, fundus images, hyperparameter optimization, improved Whale optimization, Kolmogorov-Arnold network, pre-trained models, ShuffleNet V2, transfer learning

## Abstract

**Introduction:**

Diabetic retinopathy (DR) is an inflammatory condition affecting the retina caused by elevated and unregulated blood glucose levels. On a global scale, it is a contributing factor to vision impairment. Several deep learning (DL) methods use retinal images to identify DR severity. However, a significant improvement is required to assist medical professionals in recognizing DR in its early phases.

**Methods:**

Thus, the author introduced a method based on the DL technique to determine the DR severity grades using retinal images. A ShuffleNet V2 model with vision transformers’ (ViT) attention mechanism was used to extract the features. An improved Whale optimization method (IWO) was used to fine-tune the feature extraction model. We employed a convolutional Kolmogorov-Arnold Network to categorize the DR severity using the extracted features. The EyePACS dataset was utilized to train the proposed DR severity grading model using a five-fold cross-validation strategy. We generalized the model on the Messidor-2 dataset.

**Results:**

The findings revealed an average accuracy of 93.84% on the MESSIDOR-2 dataset, demonstrating a substantial improvement in detecting DR using the fundus images.

**Discussion:**

Furthermore, the model demands minimal processing resources to generate the outcomes, leading to the deployment of the proposed DR severity detection model in healthcare facilities with limited computational resources.

## 1 Introduction

A key challenge in the medical field is offering a diagnostic tool for ocular diseases utilizing retinal images (1). Ocular diseases encompass a wide range of conditions and disorders that have an adverse effect on visual acuity or the eye’s ability to function normally (2, 3). Fundus abnormalities account for the vast majority of cases of blindness in individuals across the globe. The World Health Organization specifies that a plasma glucose level of more than 7.0 mmol/L constitutes a diagnosis of diabetes mellitus in a patient (4). The most prevalent eye diseases caused by diabetes mellitus include diabetic retinopathy (DR) and cataracts (5, 6). In most cases, both eyes will be affected by DR (5). Long-term diabetes increases the risk of DR (7, 8). Blindness may result from untreated diabetic retinopathy. Long-term high blood sugar can allow fluid to build up in the lens that regulates eye focus (9). As a result, eyesight is affected by irregular lens curvature. The fundus images are examined to identify and evaluate the severity levels of DR. Microaneurysms, hemorrhages, hard exudates, and other signs of ocular bleeding and exudation are used to determine the severity levels (10, 11). DR may be diagnosed and graded by retinal morphological abnormalities caused by diabetes. Fundus imaging aids in the early detection of DR with the assistance of computer-aided design (CAD) (12). Fundus images are preprocessed, segmented, analyzed, and graded based on the severity levels of DR (13).

Recent developments in computer vision offered multiple techniques for capturing retinal images (14). It supports clinicians and healthcare professionals in identifying complex retinal and optic nerve diseases. The introduction of autofocusing systems minimized human interaction in aligning the aperture and exposure settings (6). Fundus cameras and Optical Coherence Tomography are widely applied to capture retinal images. In medical diagnostics, healthcare systems use artificial intelligence (AI) applications to provide effective treatments (15). In ophthalmology, AI is attracting a lot of attention in identifying sensitive and difficult-to-diagnose disorders. It assists ophthalmologists in diagnosing DR using complex fundus images. DL is a subset of AI algorithms based on artificial neural networks (16). DL model can learn the data’s latent and intrinsic relations mathematically. It generates relevant features directly from the data. It can scale better as data expands than classic machine learning (ML) algorithms (17). It can detect a range of eye-related disorders in order to enhance prediction accuracy (18). In addition, observing retina images enables the effective detection of choroidal abnormalities, hemorrhage, vascular defects, and glaucoma (19). Thus, AI may offer a reliable application for healthcare centers to identify DR in the initial stages and reduce the disease’s severity.

An automated DR severity grading model can be built using Convolutional neural networks (CNN) and other advanced DL techniques. Substantial computational resources are required to train the CNN models to deliver a reasonable outcome (19–21). The annotation process is necessary to label the medical images to train the AI techniques. However, it is challenging, expensive, and error-prone. The training duration can be minimized by transferring the weights of the pre-trained models to the CNN model to perform a complex image processing task (21). Due to its hierarchical convolutional structure, CNNs may extract low-level and mid-level spatial characteristics such vascular shape, localized texture, and lesion borders. However, these models are limited in capturing broader global context. In recent years, Vision Transformers (ViTs) have emerged as a powerful computer vision paradigm due to its self-attention mechanism, capturing long-range dependencies and global contextual relationships across an image (22). This architecture can evaluate interactions between distant retinal areas including the optic disc, macula, and peripheral vasculature. Using the spatial distribution and interrelationships of microaneurysms, hemorrhages, and exudates across the fundus image, it can support the classifier in grading DR severity. Furthermore, the attention mechanism guarantees that clinically noteworthy global patterns are included into the decision-making process, presenting diagnostically crucial DR features. A hybrid CNN–ViT architecture can offer strong localized representations and the transformer’s attention mechanism integrates these features into a holistic representation of the retinal image (22). Hybridization increases feature richness and resistance to contrast variation, illumination artifacts, and device-specific noise (22). This combination improves DR severity classification in diverse clinical contexts.

The selection of the classifier head is as critical as hybrid feature extraction. The majority of DR severity grading techniques use dense fully connected layers or gradient boosting, which are successful in simpler classification tasks. However, these classifier encounter challenges in modeling intricate multivariate relationships in multiclass DR prediction. Feature-engineering-dependent gradient boosting algorithms typically fail to capture deep features’ non-linear hierarchical interactions, and dense layers are prone to overfit, particularly when there is a class imbalance. Therefore, an effective classifier should provide flexibility in function approximation and robust generalization to novel data.

A robust and adaptable model is required to serve a wide range of global populations. The presence of imbalances in the distribution of various severity levels of DR within datasets may lead to significant challenges (23). Addressing data imbalance and mitigating biases are crucial in developing an AI-powered DR severity classification. Healthcare facilities require a DL-based model to identify diabetic retinopathy and reliably categorize its severity with limited resources. The lack of transparency in DL models is a significant challenge for clinicians in interpreting the rationale of a specific decision (23).

Automating the DR severity classification can assist clinicians in rendering effective service to patients. It supports the individuals in recovering from the illness. Medical error can be minimized by providing optimal results. The current state of knowledge in DL-based DR severity detection lacks the development of lightweight models that effectively maintain a tradeoff between accuracy and computing power. Resource-constrained devices benefit from lightweight models, including mobile devices, edge devices, and low-power machines. These devices are specifically engineered to optimize memory usage and processing capabilities, rendering them ideal for real-time or on-device computational tasks. Implementing lightweight models in the context of DR screening offers a cost-effective solution by eliminating the need for costly hardware or cloud-based processing. This facilitates the accessibility and long-term sustainability of regular evaluations. Thus, there is a scope for developing lightweight multi-class classification models using complex images. This motivates the researcher to build a DL-based DR detection framework using the fundus images. Thus, this study proposes a DR severity classification model using an effective feature extraction technique. The key contributions of the proposed research are:

- Hybrid CNN-VITs-powered feature extraction technique using improved Whale optimization (IWO) algorithm.
- DR severity level identification using convolutional Kolmogorov-Arnold Networks (cKANs).

The novelty of this study lies in combining CNN and ViTs’ attention mechanism to extract DR features from the fundus images. In addition, the introduction of cKANs as the classification head. Unlike standard Kolmogorov-Arnold Networks (KANs), cKANs integrate convolutional processing that enables the proposed DR severity grading model to handle structured spatial features directly before decomposing them into univariate functional approximations, rendering it suitable for DR severity classification rather than standard dense classifiers and gradient boosting algorithms.

The remaining part of the proposed study is divided as follows: Section 2 outlines the features and limitations of the existing DR detection models. The process of data acquisition, image preprocessing, feature extraction, and image classification techniques are described in Section 3. The experimental results are outlined in Section 4. Section 5 provides the features of the proposed study in detecting DR using fundus images. Lastly, the study’s contributions, challenges, and future directions are discussed in Section 6.

## 2 Literature review

The existing screening methods for DR include detection and grading. Diabetic retinopathy is detected through binary classification, and grading involves identifying and labeling infected areas based on the severity. In DL-based DR detection, CNN models were trained on retinal fundus images to identify DR lesions by recognizing edges and textures in lower layers and abstract characteristics in higher levels (23). VGGNet, ResNet, and DenseNet have been applied from scratch or with pre-trained weights on massive datasets like ImageNet and fine-tuned on DR datasets to increase performance. To enhance the training phase, the generative adversarial network may be used to generate synthetic retinal images (24). It improves DL model resilience to imaging circumstances and diversifies training data. Attention processes in CNN designs may highlight diabetic retinopathy lesion-associated retinal images, enhancing detection accuracy. Domain adaptation approaches may transfer information from the public to local clinical datasets, boosting model performance on domain-specific data.

Alharbi and Alhazmi (4) highlighted the prevalence and risk factors of diabetic mellitus across Saudi Arabia. This study’s findings revealed an exponential growth of DR among the Saudi population. DR can be identified using visual acuity, pupil dilation, and tonometry in the traditional system. However, patients may be negatively impacted by the additional time and effort required for these tests. In addition, the disease’s microscopic nature causes challenges in detecting DR in the initial stages. Mahmoud et al. (7) introduced a DR detection technique using a patch division-based DenseNet model. They employed DenseNet 201 and neighborhood component analysis for feature extraction and selection. Cubic support vector machine (SVM) was used for multi-class classification. Kassani et al. (14) applied multiple CNN models for DR image classification.

Li et al. (19) introduced a DL-based model using the ensemble approach. Ramos et al. (20) used a supervised learning approach to detect the severity levels of DR. Orlando et al. (23) proposed a domain knowledge-based DL approach for detecting DR. They employed the image-to-text mapping feature space to find the features of the images. A candidate detection strategy was used to extract the intricate patterns from the fundus images. Random Forest classifier was used to classify the images. Similarly, the studies (12, 16, 24–27) proposed DL-based models for classifying the severity levels of DR. Most studies employed Messidor-2 (28, 29), APTOS-2019 (30), and EyePACS (31) for evaluating the performance of the DR detection models. Recent studies [] proposed hybrid feature extraction mechanism, supporting the classifiers in achieving optimal classification accuracy. The characteristics of the existing studies is described in Table 1.

**Table 1:** Features and limitations of existing literature.

Authors	Dataset	Features	Limitations
Mahmoud et al. (7)	APTOS -2019	Patch-based DenseNet 201, NCA feature selection, and Cubic SVM were employed.	Cubic SVM demands substantial computing resources.
Orlando et al. (23)	DiaRetDB1 and e-Ophtha	Employed ensemble learning approach with candidate detection strategy.	The size of the datasets was low. The performance may vary in real-time datasets.
Li et al. (19)	MESSIDOR-2	An improved Inception-V4 ensembling approach was used.	Trained on private datasets. Grader biases may influence the model’s performance.
Zhang et al. (24)	APTOS-2019 and EyePACS	Source-free transfer learning was employed to identify DR using unannotated images.	The model demands substantial computational resources.
Kassani et al. (14)	APTOS-2019	Modified Xception architecture was used for feature extraction.	The model was validated using a single dataset.
De La Torre et al. (25)	EyePACS	The layer-wise relevance propagation method was used for DR grading.	Limited to binary classification.
Gambhir et al. (51)	APTOS-2019	ShuffleNet V2 with Smooth L2 loss function was used to extract features.	The model was not fine-tuned, and its performance may vary in clinical settings.
Lu et al. (52)	MESSIDOR-1	MobileNet V2 and ResNet-50-based feature extraction.	The model was trained with a limited set of samples.
Bapatla and Harikiran (53)	IDRiD, MESSIDOR, and Kaggle datasets	A hybrid-optimized DL network was used to classify DR severity.	The model encompassed ResUnet and ShuffleNet V2 models that require substantial computational power.
Wahab Sait (49)	APTOS-2019 and EyePACS	MobileNet V3-Small model was used for the feature extraction.	Extensive image preprocessing was required.
Ashwini and Dash (54)	IDRid and EyePACS	A multi-resolution-based decomposition of discrete wavelet transform was used for DR grading.	The shortcomings of discrete wavelet transform may reduce the model’s performance.
Tian et al. (26)	DDR, MESSIDOR, APTOS-2019, and EyePACS	Fine-grained attention and knowledge-based collaborative network-based feature extraction.	The model may produce complexities during implementation.
Nazih et al. (22)	Fine-grained annotated DR Dataset	ViT with global attention and class balancing techniques.	Due to overfitting and high computational demand, the model’s performance may degrade on smaller datasets.
Chintamreddy and Seshasayee (27)	EyePACS	Hybrid convolutional and ViT architecture.	Details of training setup and imbalance handling are not explicitly discussed.
Kakade and Kakade (55)	APTOS 2019	Hybrid CNN-ViT architecture with Squeeze Excitation block.	Limited details on external validation.
Ikram and Imran (56)	APTOS 2019	Hybrid CNN-ViT fusion network, integrating explainability using Grad-CAM visualization.	Fusion complexity can increase latency. Effective fusion mechanisms demand substantial computational overhead.
Zhang et al. (48)	EyePACS and APTOS 2019	Hybrid CNN-ViT feature extraction mechanism with classification head.	Limited data size / class imbalance.
Addya et al. (32)	MESSIDOR	Hybrid ConvNeXt + KAN architecture, enabling nonlinear function approximation beyond traditional CNN layers.	The individual impact of each KAN block is not thoroughly quantified.
Bashir et al. (33)	APTOS 2019	Applied B-spline–based enhancement to improve contrast, edge sharpness, and visibility of DR lesions.	Focuses only on image enhancement, without integrating a modern deep learning classifier or end-to-end pipeline.

Recent studies (32, 33) indicate the importance of KAN in medical image categorization / classification. A number of characteristics make KAN-inspired structures ideal for medical image categorization. In contrast to traditional fully connected layers that depend on static linear transformations, KAN modules use spline-based functional approximations, mimicking the nonlinear, smooth fluctuations observed in medical imaging. This characteristic is particularly advantageous for detecting minute intensity gradients, edge transitions, and localized structural abnormalities indicative of early-stage lesions. In addition, KANs accomplish a great deal of expressive power with a limited number of parameters, which helps to alleviate the problem of overfitting that often occurs in clinical research with limited or unbalanced datasets related to medicine. As medical features are diverse and multimodal, lesion appearance may vary with anatomy, imaging circumstances, and pathology severity, their capacity to approximate complicated multivariate mappings is ideal. Together with visual explanation methods like Grad-CAM, the interpretability of spline-based modifications increases transparency, which in turn helps physicians comprehend feature behaviors and decision boundaries. The combined features of KAN-inspired models provide them a potential avenue for developing robust, data-efficient, and clinically interpretable medical imaging systems.

The development of accurate and robust diagnostic systems is impeded by a number of knowledge gaps, even though the current DR detection models have made tremendous progress. Variations in imaging factors, demographics, and medical condition features may prevent DL models from generalization. The existing models provide limited interpretability, making it hard for medical professionals to understand model predictions. DL algorithms generate predictions without observing uncertainty, which may affect clinical decision-making. In addition, data and annotation biases may impair model performance across diverse demographic groups. There is a demand for effective strategies to address the knowledge gaps in DL-based DR detection.

## 3 Materials and methods

**Figure 1:**
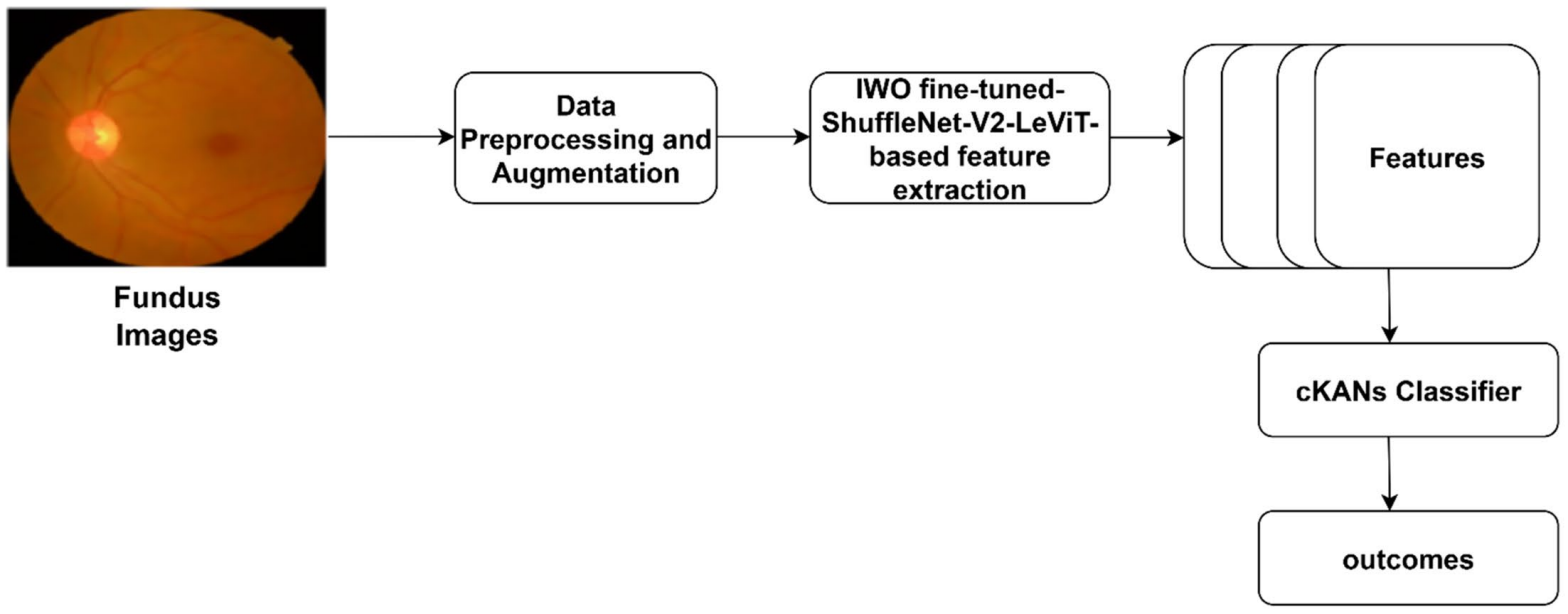
Proposed DR detection framework.

In this study, we propose a DL technique for predicting DR. The multiple phases of the proposed research are presented in Figure 1. A preprocessing task is performed to enhance image quality and reduce noise amplification. We optimize the feature extraction pipeline (shuffleNet-V2-LeVit) using the IWO algorithm. The cKANs classifier is optimized using Bayesian Optimization with Hyperband (BOHB). Figure 1 highlights the proposed DR severity classification approach.

### 3.1 Dataset description

**Figure 2:**
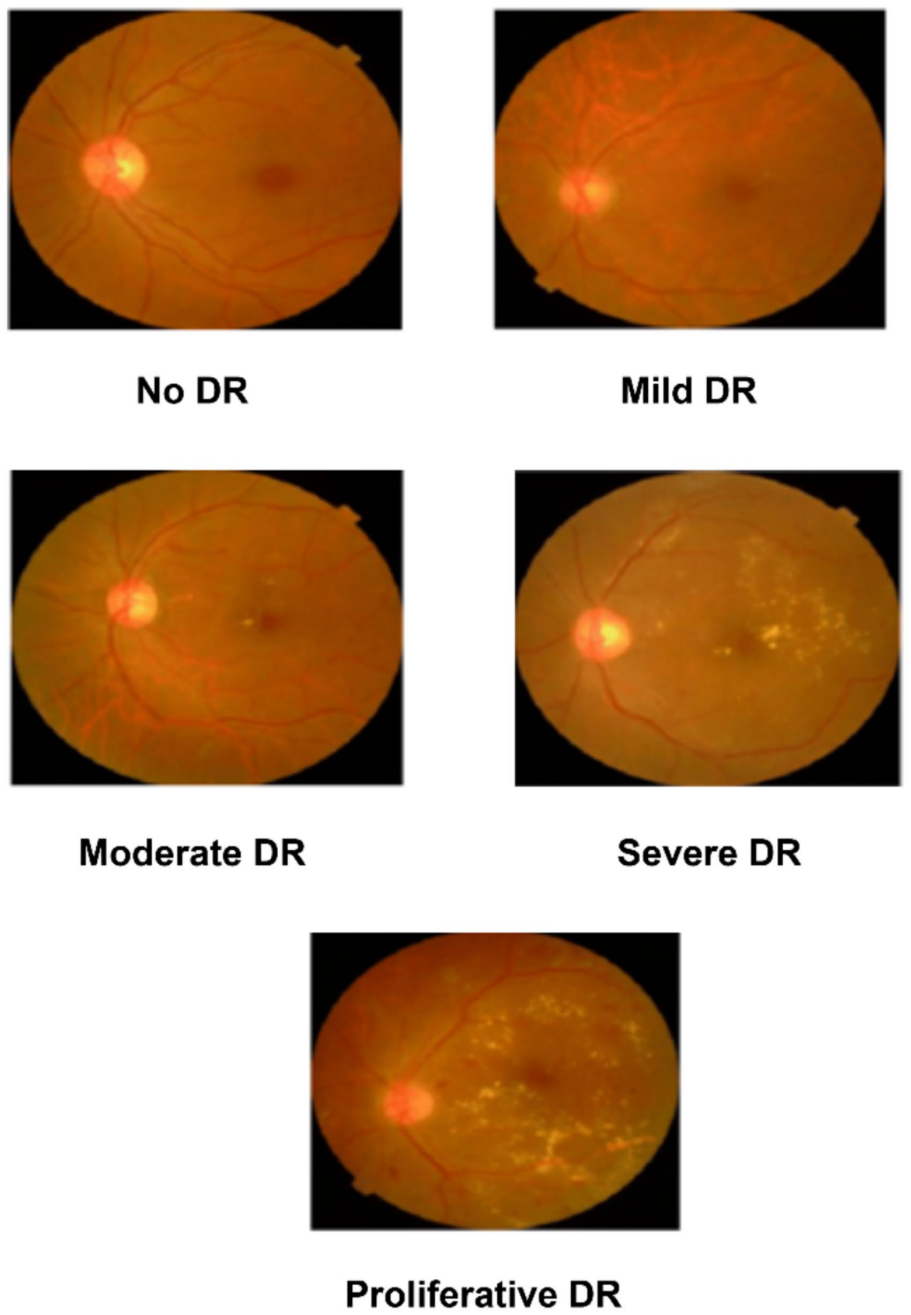
Sample DR severity images.

MESSIDOR-2 (28, 29) and EyePACS (31) datasets are the publicly available benchmark DR datasets. The proposed DR severity grading model is trained and tested using these datasets. The EyePACS dataset contains a large collection of high-resolution fundus images. It covers a broader range of DR images across the globe. The dataset providers employed clinicians to rate the presence of DR in each image. The EyePACS dataset is a competition dataset that contains diverse fundus images. The diversity in the dataset supports the proposed study in predicting the severity of the DR with optimal accuracy. MESSIDOR – 2 includes a total of 1748 images. The images are not annotated and are available in different resolutions. The images are classified into five grades: Normal (0), Mild (1), Moderate (2), Severe (3), and proliferative diabetic retinopathy (PDR) (4). The dataset attributes are listed in Table 2. Figure 2 shows the sample images of DR severities.

**Table 2:** Datasets attributes.

Grades	MESSIDOR-2	EyePACS
Normal (0)	1,017	25,783
Mild (1)	270	2,459
Moderate (2)	347	5,304
Severe (3)	75	878
PDR (4)	35	703

### 3.2 Data preprocess and augmentation

**Figure 3:**
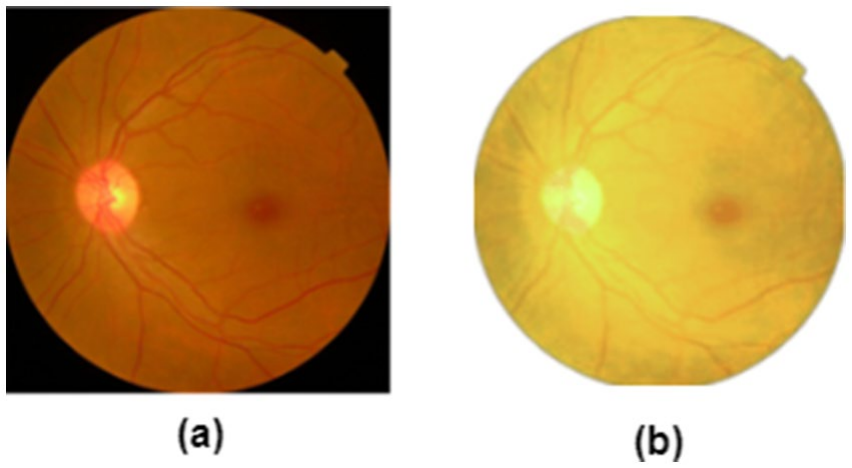
**(a)** Before enhancement **(b)** after enhancement.

Data preprocessing is beneficial for training stability, playing a crucial role in enhancing model generalization to unseen data (34, 35). The standardization of the fundus images minimizes dataset bias and domain shift. Noise suppression and contrast enhancement improve the signal-to-noise ratio. Using this strategy, CNN filters capture lesion edges and textures while guiding ViTs attention maps toward clinically relevant retinal regions, emphasizing pathology rather than acquisition artifacts. Additionally, the preprocessing step enhances the visibility of minority-class lesions, leading to reliable predictions. Thus, we apply contrast-limited adaptive histogram equalization (CLAHE) to overcome noise amplification. Let *I* be a retinal image and C be the CLAHE function. Equation 1 outlines the mathematical expression for enhancing the image contrast (36).


$$I=\sum_{i=1}^{n}C(f_{i})$$ (1)

where i and n are the number of images in the datasets. Figures 3a,b presents the normal and enhanced images.

Furthermore, the pixel values of the images are scaled in the range [−1,1]. The photometric distortions are applied to enable random changes in the images to increase the number of images with unique color, contrast, and brightness. It supports the training phase of the ShuffleNet V2 using IWO. Equation 2 presents the expressions for the data augmentation (37).


$$D(I)=\sum_{i=1}^{n}\mathrm{PD}(f_{i})$$ (2)

where D is the dataset, and PD is the photometric distortions function.

To train and evaluate the proposed DR classification model, we used the publically accessible EyePACS dataset. A five-fold stratified cross-validation strategy was applied in order to provide a reliable and unbiased evaluation of the performance of the model, maintaining the proportional distribution of DR severity classes throughout the training and testing sets. Through this approach, the possibility of data imbalance and leakage is reduced. Four folds were utilized for training, while the fifth fold serve as the test set. We applied comprehensive image augmentation techniques exclusively to the training folds in order to enhance the diversity of the training data and mitigate overfitting. Random geometric and photometric modifications were included into the augmentation process. These transformations included variations in brightness, contrast, and saturation, as well as rotations (with a tolerance of ±15 degrees), horizontal and vertical flips, scaling, and translations. Through the use of these enhancements, realistic variations in imaging settings were simulated, minimizing the possibility of overfitting and improving the resilience of feature extraction. The test fold remained unaltered in order to accurately reflect the clinical settings. In addition, in order to evaluate the model’s generalizability, we conducted an independent external validation on the MESSIDOR-2 dataset. In this validation, 40% of the images were selected at random in order to generate a test set. This methodology allowed for a comprehensive evaluation of the model’s resilience, supporting its application in real-world diagnostic procedures.

### 3.3 Feature extraction

**Figure 4:**
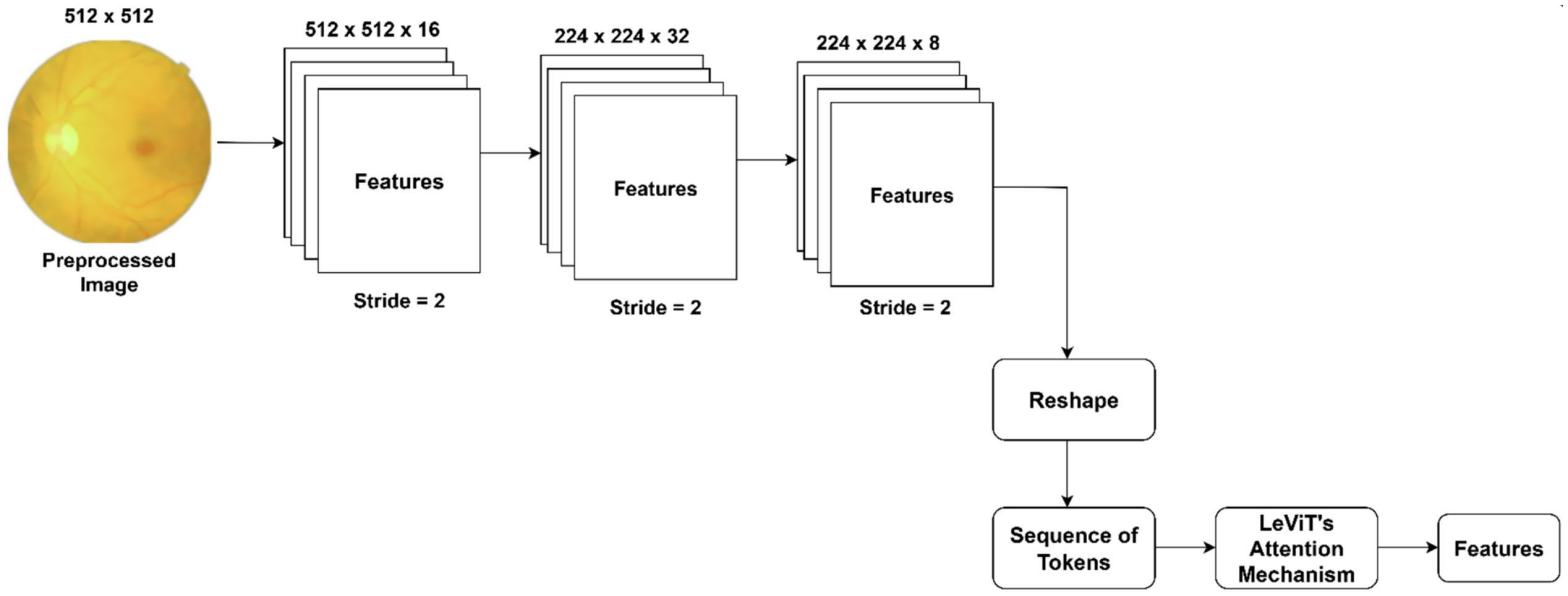
Proposed feature extraction approach.

The proposed DL model typically utilizes a variety of image features in order to find the severity level. Microaneurysms are the small round red dots caused by minor blood vessel damage. Exudates are yellow or white lesions associated with damaged blood vessels. Hemorrhages are red spots, indicating bleeding from damaged blood vessels. The feature extraction technique detects and localizes these conditions based on size, shape, and appearance. The proposed feature extraction framework utilizes serially connected ShuffleNet V2-LeViT’s attention mechanism, capturing hierarchical retinal features at multiple levels. Using its computational efficiency and channel split-shuffle strategy, ShuffleNet V2 extracts low-level vascular patterns, microaneuryms, and local textural features with limited computational resources. We employ ShuffleNet V2-50 backbone in this study to classify the images. In order to generate long-range dependencies between spatially distant retinal regions, the extracted ShuffleNet V2’s features are passed to LeViT’s attention in order to produce a final set of features. Figure 4 reflects the architecture of the proposed feature extraction pipeline.

Initially, the fundus images (*I*) are processed through the ShuffleNet V2-50 backbone (38), extracting features. Equation 3 highlights the process of feature extraction.


F=ShuffleNet_V2(I) (3)

where *F* is the ShuffleNet V2’s features and *ShuffleNet_V2* is the CNN feature extraction model.

Multiple branches are used to perform elementwise operations, including ReLu, depthwise convolutions, Concat, channel shuffle, and split. The extracted features (feature maps) are processed through the LeViT’s attention mechanism. The feature maps are reshaped into a sequence of tokens as shown in Equation 4.


$$X=\text{Reshape}(F)\in {\mathbb{R}}^{N\times d}$$ (4)

where X is the sequence of tokens, N is the number of tokens, Reshape is the function transforming the features into tokens, and d is the embedding dimension.

LeViT’s attention mechanism re-weights the features based on their significance related to the entire image. Equation 5 reflects the extraction of the contextualized feature maps (Z) using the LeViT’s block, integrating residual connections and feed-forward layers. This hybrid approach offers local lesion sensitivity and global relational awareness, which are crucial for DR severity grading.


Z=LeViT(X) (5)

where *Z* is the final set of contextualized features and *LeViT* is the ViT feature extraction model.

We optimize the feature extraction process using IWO. The traditional Whale optimization algorithm (39, 40) experiences premature convergence and may end up in local optima during the optimization of the feature extraction process. To overcome this limitation, we integrate Cauchy mutation (41) into the Whale optimization processes. The long-tailed property of the Cauchy mutation technique enables the optimization algorithm to make occasional larger jumps in the search space, preventing the optimizer from stagnating around suboptimal solutions. Thus, the update mechanism of Whale optimization technique is modified using the Cauchy mutation (41) to minimize the computation time for locating the features.

IWO enhances the traditional Whale optimization using multiple strategies. It supports the proposed framework for extracting meaningful features. Equation 6 shows the search space with the whale and the prey (41).


$$\vec{S}=|\vec{C}*{\vec{W}}_{\mathrm{rand}}(t)-\vec{W}(t)|$$ (6)

where $\vec{S}$ is the search space, $\vec{C}$ is the co-efficient vector, ${\vec{W}}_{\mathrm{rand}}$ is the randomly selected prey, $\vec{W}$ is the current individual prey. Equation 7 shows the mathematical expression to denote the whale in the next iteration for locating the prey (41)


$$\vec{W}(t+1)={\vec{W}}_{\mathrm{rand}}(t)-\vec{A}*\vec{S}$$ (7)

where $\vec{A}$ is the co-efficient vector when ∣A∣<1, the whales choose either bubble-net attacking or encircling prey for locating the prey (features). In contrast, when ∣A∣≥1 , the whale captures the prey by updating its current position.

The updating mechanism of IWO plays a crucial role in generating the feature maps. It encompasses three strategies: encircling prey, bubble-net attacking, and capturing prey. Equation 8 shows the chaotic initial population of images (41, 42).


$$Ch_{j+1}=\{\begin{array}{c} \frac{Ch_{j}}{0.7},Ch_{j}<0.7\\ \frac{1-Ch_{j}}{0.3},Ch_{j}\geq 0.7 \end{array}\}$$ (8)

where $Ch_{j}$ is the chaotic vector contains $\{Ch_{1},Ch_{2},\ldots \ldots ,Ch_{S}\},$ S is the search space. Using the chaotic vector, Equation 9 represents the whale, ${\vec{W}}_{j}$ (41)


$${\vec{W}}_{j}={\vec{W}}_{\mathrm{lb}}+({\vec{W}}_{\mathrm{ub}}-{\vec{W}}_{\mathrm{lb}}).\vec{\mathrm{Ch}}$$ (9)

where ${\vec{W}}_{\mathrm{lb}}=\{W_{1}^{\mathrm{lb}},W_{2}^{\mathrm{lb}},\ldots \ldots ,W_{S}^{\mathrm{lb}}\},$ ${\vec{W}}_{\mathrm{ub}}=\{W_{1}^{\mathrm{ub}},W_{2}^{\mathrm{ub}},\ldots \ldots ,W_{S}^{\mathrm{ub}}\}$ are the lower and upper boundaries for the whales to move forward and backwards for selecting the features. To maintain the tradeoff between the exploration and exploitation functionalities of the whales, Equation 10 is employed (42).


$$A=2-2\frac{e^{{\left(\frac{t}{t_{\max}}\right)}^{m}}-1}{e-1}$$ (10)

where m is the curve smoothness value of A and t is the iteration value.

Equation 11 indicates the inertia weight for generating the best solution using the position of the whales (42).


$$W_{t}=|\cos(\frac{\mathrm{lt\pi}}{t_{\max}})|$$ (11)

where $W_{t}$ is the weight and l be the cycle size of the weight function.

In the current environment, the whales can face challenges in addressing the local optimum problem. To overcome the problem, the Cauchy mutation is introduced with inertia weight. It assists the whales in producing a mutant vector to find an optimal solution using the Cauchy distribution. Equation 12 shows the Cauchy mutation vector (42).


$${\vec{\mathrm{WV}}}_{i,g}^{i}=\{\begin{array}{c} \begin{array}{l} \text{Cauch}y_{i}^{j}(W_{\text{best},g}^{i},0.1),\text{if}\;\\ \text{Cauch}y_{i}^{j}<\text{crossove}r_{i,g}^{j}\text{or}\;j=j_{\text{Cauchy}} \end{array}\\ W_{i,g}^{j},\text{Otherwise} \end{array}\}$$ (12)

where $\text{Cauch}y_{i}^{j}$ is the Cauchy distribution function, $W_{\text{best},g}^{i}$ is the whale, $WV_{i,g}^{i}$ is the mutant vector, and i, j, and g are the ranges in which the whales locate the features. Equation 13 presents the update mechanism for the whales using the mutant vector.


$$\vec{W}(t+1)=\{\begin{array}{c} \mathrm{wt}*{\vec{\mathrm{WV}}}_{i,g}^{i}-\vec{A}*\vec{S},P<0.5\\ \mathrm{wt}*{\vec{\mathrm{WV}}}_{i,g}^{i}+|{\vec{\mathrm{WV}}}_{i,g}^{i}-W_{i,g}^{j}|*e^{\mathrm{bl}}*\cos(2\mathrm{\pi l}),P<0.5 \end{array}\}$$ (13)

where b and l are constant and random numbers, respectively.

Furthermore, the optimal-based feedback mechanism is used to overcome the uncertainty in updating the whale’s position during the exploratory process. Equation 14 shows the mathematical form for updating the whale’s location (42).


$${\vec{W}}_{\mathrm{new}}=\{\begin{array}{c} \lambda {\vec{W}}_{\mathrm{rand}}+(1-\lambda )\vec{\mathrm{WV}}+C({\vec{W}}_{\mathrm{rand}}-\vec{\mathrm{WV}}),\mathrm{pl}>\text{threshold}\\ {\vec{W}}_{\mathrm{rand}},\mathrm{pl}\leq \text{threshold} \end{array}\}$$ (14)

where λ and pl. are random values between 0 and 1, and C is the random value in the range [−1,1].

### 3.4 DR prediction model

The cKANs framework (43–47) is an emerging class of neural architectures. It is parameter efficient compared to existing machine learning models. It combines the representation of learning capabilities of convolutional networks with the functional approximation potential of Kolmogorov-Arnold superposition theory (43–47). Instead of representing feature vectors as monolithic inputs, cKANs break them down into multiple one-dimensional functional pathways, represented by smooth spline basis functions. This type of design is sufficient for the network to encode localized variations in feature intensity and spatial transitions generally observed in retinal pathology. A key distinction from dense layers is that cKANs do not depend on global matrix multiplications, rather, they accumulate expressivity through successive nonlinear compositions of simpler univariate units. This results in a drastic reduction of parameter redundancy and increases the generalization ability of the model with limited medical imaging data. The multivariate synthesis stage then aggregates these localized representations into diagnostically meaningful patterns, providing fine discrimination between DR severity levels. By capturing hierarchical relationships between lesion attributes, such as the progression of sizes, the increase in density, and the distributed lesion clusters, cKANs provide a more anatomically aligned decision process than traditional fully connected architectures. First, each input channel is first transformed through cubic B-spline basis functions, allowing for smooth nonlinear approximation and fine-grained variations in features extracted from images. This is especially useful in medical imaging, where small changes in intensity, and small patterns in the structure, are crucial for diagnosis. The combination of these spline-expanded features in the form of spline combinations to form the higher-order interactions without increasing the parameterization size is carried out by the multivariate mixer. Unlike fully connected layers, which are based on global linear projections, cKANs can perform expressive nonlinear modeling with far less learnable parameters and better resistance to overfitting. In the context of DR grading, cKANs are particularly suitable since they successfully model the smooth intensity gradients, lesion boundaries and spatial continuity of microaneurysms, hemorrhages, and exudates. This enables the classifier to separate adjacent DR grades that are only slightly separated into morphologic changes.

**Figure 5:**
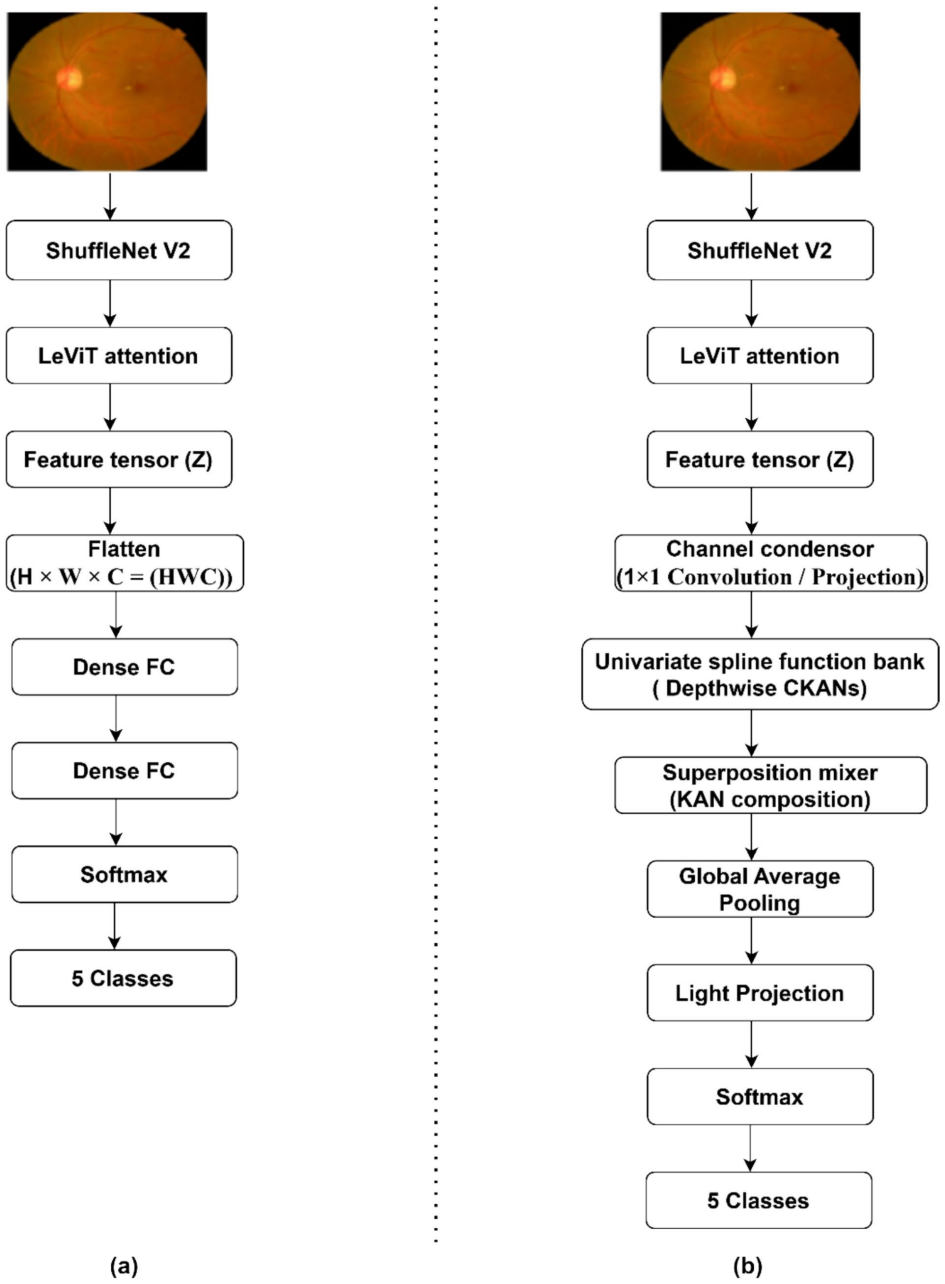
Comparison of classifier heads for DR severity grading. **(a)** Standard CNN–ViTs pipelines typically flatten feature maps and use fully connected (FC) layers, which increases parameter coupling and may reduce spatial interpretability. **(b)** The proposed approach replaces the dense head with a cKANs module, where channel condensation, univariate spline approximation, and superposition mixing preserve structured representations and improve parameter efficiency for multi-class DR grading.

Furthermore, cKANs dramatically reduce the number of learnable parameters when compared to dense layers, which helps reduce overfitting, a crucial benefit given the class imbalance and small number of high-quality DR annotations that are commonly found in medical datasets. Their ability to learn smoother, more clinically meaningful feature mappings ultimately enhances the network’s ability to discriminate between adjacent DR grades, where visual differences are nuanced and depend on lesion distribution rather than coarse global patterns. Figures 5a,b highlight the significance of the cKANs module, comparing standard CNN-ViTs against the proposed CKANs-driven classification.

Through functional decomposition, cKANs (43–47) reduce overfitting. The cKANs module transforms the feature maps into univariate function spaces, yielding the final prediction. The channel condenser reduces channels and features for univariate paths. Univariate function bank is a depthwise seperable cKANs layer. Each channel is processed by a depthwise convolution followed by a learnable spline attention function, approximating univariate function per channel. The superposition mixer forms finite superposition of the univariate outputs in order to approximate the multivariate map. In pooling and projections, global average pooling is used to reduce spatial axes. Subsequently, the softmax function produces multiclass probabilities. Table 3 summarizes the comprehensive tensor flow through the CKANs module.

**Table 3:** Stage wise tensor flow via the CKANs module.

Stage	Operation	Description	Output from the operation	Tensor Shape
0	Input from ShuffleNet V2 + LeViT	Contextualized feature maps	Contextualized feature maps (*Z*) with batch size (*B*)	$Z\in {\mathbb{R}}^{B\times 14\times 14\times 256}$
1	Channel condenser (1 × 1 conv / projection)	Reduces channel redundancy before spline approximation	Channel-condensed feature maps (*Z_c_*)	$Z_{c}\in {\mathbb{R}}^{B\times 14\times 14\times 128}$
2	Univariate spline function bank (depthwise cKAN)	Channel-wise cubic B-spline functions (order 3, 8 knots)	Channel-wise spline-expanded features (*U*)	$U\in {\mathbb{R}}^{B\times 14\times 14\times 128}$
3	Superposition mixer (1 × 1 conv)	Kolmogorov–Arnold-style composition; reconstructs multivariate mapping	Multivariate composition output (*M*)	$M\in {\mathbb{R}}^{B\times 14\times 14\times 256}$
4	Global average pooling (GAP)	Aggregates spatial information	Globally pooled descriptor (*g*)	$g\in {\mathbb{R}}^{B\times 256}$
5	Linear layer + Softmax	Maps to DR severity classes (0–4)	Final probability vector output (*p*)	$p\in {\mathbb{R}}^{B\times 5}$

To optimize the feature-extraction pipeline, each candidate hyperparameter configuration is evaluated using a quantitative fitness function as shown in Equation 15, reflecting the discriminative quality of the extracted features. Using this function, classification performance is maximized while penalizing configurations with excessive computational cost, ensuring that the final model remains lightweight and deployable.


$$ℱ=\alpha (1-\text{ValAccuracy})+\beta \times \text{ValLoss}+\gamma \times \frac{\text{FLOPs}}{\text{FLOP}s_{\max}}$$ (15)

where ℱ denotes the fitness function, ValAccuracy represents the validation accuracy of the cKANs module, ValLoss is the validation cross-entropy loss, α , β , and γ are trade-off weights, and FLOPs measures the computational complexity of the candidate feature extractor, normalized by the maximum FLOP ( $\text{FLOP}s_{\max})$ value considered in the search. The minimization of ℱ encourages IWO to select effective hyperparameter configurations in order to maximize discriminatory power of the extracted features, maintain stable convergence behavior, and preserve the lightweight constraints for deployment on low-resource settings.

This composite fitness formulation enables IWO to jointly evaluate representational quality and computational efficiency, making it more suitable than accuracy-only objectives for real-time DR screening applications. A learnable non-linear activation function is used to integrate each feature. The total training objective $O_{\text{total}}$ is presented in Equation 16.


$$O_{\text{total}}=O_{\text{pred}}+\lambda (\mu_{1}\sum_{O=0}^{O-1}{|\phi_{O}|}_{O}+\mu_{2}\sum_{O=0}^{O-1}S(\phi_{O}))$$ (16)

where $O_{\text{pred}}$ is the severity identification, $\mu_{1}$ and $\mu_{2}$ are magnitudes, λ is the regularization magnitude, and $\phi_{O}$ is the entropy.

We employed Bayesian Optimization HyperBand (BOHB) to fine-tune the cKANs model. In addition, we introduced an early stopping strategy, quantization-aware training (QAT), and SHapley Additive exPlanations (SHAP) values to enhance the model’s performance. QAT was used in the training phase in order to prepare the model for deployment in a resource-constrained environment. SHAP values were used to gain insights into the model’s behavior.

### 3.5 Performance evaluation

The benchmark metrics, including accuracy, precision, recall, F1-score, Matthews Correlation Coefficient (MCC), and Cohen’s Kappa, are used to measure the classification efficiency of the frameworks. Equations 17–22 present the mathematical form of the evaluation metrics.


$$\text{Accuracy}=\frac{\mathrm{TP}+\mathrm{TN}}{\mathrm{TP}+\mathrm{TN}+\mathrm{FP}+\mathrm{FN}}$$ (17)

$$\text{Precision}=\frac{\mathrm{TP}}{\mathrm{TP}+\mathrm{FP}}$$ (18)

$$\text{Recall}=\frac{\mathrm{TP}}{\mathrm{TP}+\mathrm{FN}}$$ (19)

$$F1-\text{Score}=2\times \frac{\text{Precision}\times \text{Recall}\;}{\text{Precision}+\text{Recall}}$$ (20)

$$\mathrm{MCC}=\frac{\mathrm{TP}\times \mathrm{TN}-\mathrm{FP}\times \mathrm{FN}}{\sqrt{\left(\mathrm{TP}+\mathrm{FP})(\mathrm{TP}+\mathrm{FN})(\mathrm{TN}+\mathrm{FP})(\mathrm{TN}+\mathrm{FN}\right)}}$$ (21)

$$\text{Cohe}n^{\prime }s\;\text{Kappa}=\frac{P_{o}-P_{e}}{1-P_{e}}$$ (22)

where TP is the true positives, representing the cases where the model correctly predicts the positive class, TN is the true negatives, indicating the cases where the model correctly predicts the negative class, FP is the false positives, representing the cases where the model predicts the positive class when the ground truth label is actually negative, FN is the false negatives, indicating the cases where the model incorrectly predicts the negative class, when the ground truth label is actually positive, $P_{o}$ is the observed agreement, and $P_{e}$ is the expected agreement.

We compute the area under the receiver operating characteristics (AUROC), precision-recall curve (AUPRC) to ensure the capability of the proposed framework in handling image complexities. Standard deviation (SD) and confidence intervals (CI) are computed to determine the statistical significance of the findings. Finally, each model’s FLOPs, parameters, and GPU speed are computed to identify the computing cost.

## 4 Results

The proposed framework is constructed using Python 3.8.0, a single NVIDIA GeForce GTX 1080Ti, Windows 10, and 16 GB RAM. We integrate CUDA and CUDNN libraries to accelerate tensor operations, optimizing the feature extraction pipeline. The hardware-software configuration was selected to demonstrate that the proposed DR severity grading model can be deployed in resource-constrained environments without substantial computational resources. We validated the proposed DR severity grading model on the MESSIDOR-2 (40%) dataset to assess generalization capability. During the training phase, we employed a batch size of 16 due to memory constraints introduced by the multi-stage pipeline. However, a batch size of 32 was used for the inference-throughput measurements, a common approach in benchmarking to balance GPU utilization and latency. Differences in the model’s performance can be attributed to architectural and methodological improvements. A customized early stopping mechanism was introduced to prevent overfitting, reducing the risk of model memorization and improving generalization. Table 3 outlines the experimental settings for the model implementation. To ensure reproducibility, we explicitly include the hyperparameters optimized using the IWO algorithm. Unlike standard grid or random search methods, IWO is a multidimensional hyperparameter search method covering convolutional kernel size, channel width multiplier, LeViT embedding dimension, attention dropout rate, learning rate and regularization terms. Table 4 summarizes the experimental settings with a clear distinction between fixed parameters, IWO-optimized parameters and parameters optimized using BOHB for the cKANs classifier.

**Table 4:** Experimental settings.

Category	Parameter	Value / search range	Optimized by
Input and preprocessing	Input image size	512 × 512	—
	Patch/feature size	224 × 224 × 3	
Backbone network	CNN backbone	ShuffleNet V2	
	LeViT attention module	Enabled	
Hyperparameters tuned by IWO	Learning rate	[1 $e^{-5},$ 1 $e^{-2}$ ]	IWO
	Weight decay	[1 $e^{-6},$ 1 $e^{-3}$ ]	
	Batch size	{16, 23}	
	ShuffleNet V2 channel width multiplier	{0.5, 0.75, 1.0}	
	Kernel size of depthwise convolution	{3, 5}	
	LeViT embedding dimension	{128, 256, 384}	
	Number of attention heads	{4, 8}	
	Attention dropout	([0.0, 0.3])	
IWO internal parameters	Population size	{20, 30, 40}	—
	Maximum iterations	{30, 50, 70}	
	Cauchy mutation scale	([0.1, 1.0])	
	Inertia weight (linear decay)	0.9 → 0.4	
Classifier (cKANs)	Architecture	Two hidden layers + output layer	BOHB
	Spline order	3	
	Basis functions	Cubic B-spline	
	Knots per spline	8	
	Hidden units	128	
	Activation	Softmax	—
Training strategy	Optimizer	Adam	
	Regularization	Dropout (0.3), Early stopping	

A five-fold stratified cross-validation protocol was used in this research to assess the performance of the model and to provide a reliable and unbiased evaluation. All the data was initially divided into five mutually exclusive folds with the original distribution of classes maintained in each fold. Each cross-validation step i = 1.0.5 was assigned a single fold as an independent test set and the rest of the folds were used to comprise the training and validation subsets. In particular, out of the four folds of model development, 90% of the images were utilized in model training and 10% in validation, with stratified sampling, ensuring that the proportions of classes in subsets remain constant. This method ensures that (i) every sample is represented once in the test set, (ii) no image is repeated in training, validation or testing in the same iteration and (iii) there is control over the class imbalance in all subsets, which minimizes bias to majority classes. The entire process of hyperparameter tuning, optimization processes, and updated model was all conducted only on the training and validation subsets, and not on the test fold until the ultimate analysis of that particular iteration. The reported performance metrics represent the mean and standard deviation across all five-test folds, providing a robust estimate of the model’s generalization capability.

**Figure 6:**
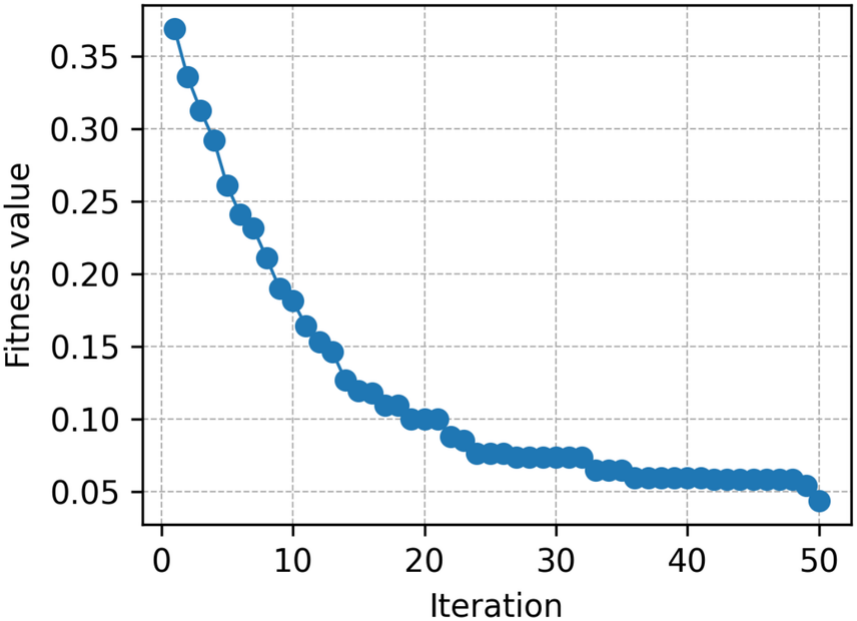
Convergence behavior of IWO.

Figure 6 indicates the convergence behavior of the IWO algorithm in 50 iterations. To justify the computational overhead introduced by the IWO algorithm, a lightweight complexity and convergence assessment is conducted, focusing on its iteration-wise performance. The computational cost of IWO primarily emerges from population updates and fitness evaluations, yielding an approximate complexity of O (P × T × F), where P is the population size, T the number of iterations, and F the feature-extraction fitness computation.

In the model implementation, IWO was configured with a moderate population size and capped iterations to prevent excessive runtime. Empirically, we observed that nearly 70–75% of the performance gain occurred within the first 15–20 iterations, after which improvements plateaued. The fitness curve shows a sharp drop in the first optimization stage (iterations 1–15), which means that the beneficial hyperparameter area of the feature extraction pipeline is quickly identified. Around 20 iterations, the curve levels off and reaches a plateau, and thereafter only slight improvements are realized. This behavior affirms that IWO is efficient convergent behavior, and does not have to undergo numerous iterations to reach an approximation of an optimum solution. The absence of significant oscillations or deviation, and the smooth monotonic trend are additional indicators that the optimization process is stable. Importantly, the rapid early-stage convergence justifies the computational overhead, as the majority of performance gains are achieved before the maximum iteration threshold. Overall, the convergence trajectory verifies that IWO can efficiently explore complex, multimodal feature-space landscapes without imposing excessive computational complexity.

The rationale for applying IWO exclusively to the feature extraction pipeline, as opposed to the classifier, stems from the distinct functional responsibilities and parameter characteristics inherent to each stage. Due to the extremely nonlinear interactions between its convolutional and attention-based components, the backbone feature extractor’s hyperparameter search space is irregular, discontinuous, and sensitive to local minima. IWO, a population-based metaheuristic, traverses the search space globally, avoids premature convergence, and adapts well to complicated, high-dimensional parameter distributions. Implementing IWO at this stage guarantees the extraction of the most informative and discriminative feature representations prior to their transfer to the classification module. The classifier, on the other hand, operates within an organized and lower-dimensional parameter space when it is implemented with cKANs. This makes the performance surface smoother and more suitable for optimization from models. Therefore, BOHB was solely used on the classifier. BOHB demonstrates superiority in continuous, well-structured search spaces by using probabilistic modeling to effectively balance exploration and exploitation. Employing BOHB for the classifier facilitates rapid convergence while preserving computational efficiency. Two factors prevented the BOHB from being applied consistently during both phases. Initially, BOHB has difficulties with the irregular, multimodal search properties of deep feature extractors, potentially leading to convergence on inferior configurations. Secondly, concurrently optimizing feature extraction and classifier components using a unified technique will substantially elevate computational expenses without ensuring enhanced performance, owing to their distinct optimization landscapes. A more efficient and empirically successful solution is provided by the two-stage heterogeneous optimization strategy, which consists of global evolutionary search (IWO) and structured probabilistic refinement (BOHB).

Table 5 outlines the findings of the five-fold cross validation experiments using the EyePACS dataset. It demonstrates the robustness of the proposed framework across different subsets of the dataset, reflecting the significance of the proposed DR severity grading model in achieving accuracy ranging between 94.34 and 97.45%. In terms of sensitivity, the model reports recall values greater than 93% for all folds, ensuring the minimization of false positives. The exceptional outcomes can be attributed to the integration of ShuffleNet V2 with LeViT attention and cKANs classifier, strengthening its applicability in clinical settings.

**Table 5:** Finding of five-fold cross-validation.

Fold	Accuracy (%) ± 95% CI half-width	MCC (%)	Kappa (%)	Recall (%)	Precision (%)	F1-Score (%)
1	97.31 ± 1.31	92.42	90.85	95.34	94.68	95.01
2	95.87 ± 1.04	90.52	89.42	95.12	95.49	95.30
3	97.45 ± 0.56	89.88	90.78	96.97	97.45	97.21
4	96.89 ± 0.69	90.45	91.52	95.47	94.87	95.17
5	94.34 ± 1.03	90.53	90.55	93.02	92.47	92.74

Table 6 outlines the performance of the proposed DR severity classification across the five severity classes. The proposed DR severity grading model achieves a strong balance between sensitivity and specificity, obtaining an average accuracy of 94.34% ± 1.03. The MCC value of 90.53% and Cohen’s Kappa of 90.55% reinforces the model’s robustness. The findings exhibit the potential of the hybrid feature extraction architecture, improving the classification performance of the cKANs model, enhancing sensitivity to subtle pathological features, which is crucial for early detection and timely intervention.

**Table 6:** Findings of the multi-class performance evaluation – EyePACS dataset.

Classes	Accuracy (%) ± 95% CI half-width	MCC (%)	Kappa (%)	Recall (%)	Precision (%)	F1-Score (%)	SD
Normal (0)	94.28 ± 1.02	90.41	89.88	93.61	92.89	93.25	0.006
Mild (1)	93.69 ± 1.29	89.56	90.77	91.90	92.07	91.98	0.007
Moderate (2)	95.08 ± 0.96	90.36	91.52	93.64	92.55	93.09	0.012
Severe (3)	94.62 ± 0.85	91.45	89.90	93.08	92.90	92.99	0.019
PDR (4)	94.02 ± 1.03	90.87	90.70	92.87	91.93	92.40	0.009
Average	94.34 ± 1.03	90.53	90.55	93.02	92.47	92.74	0.010

**Figure 7:**
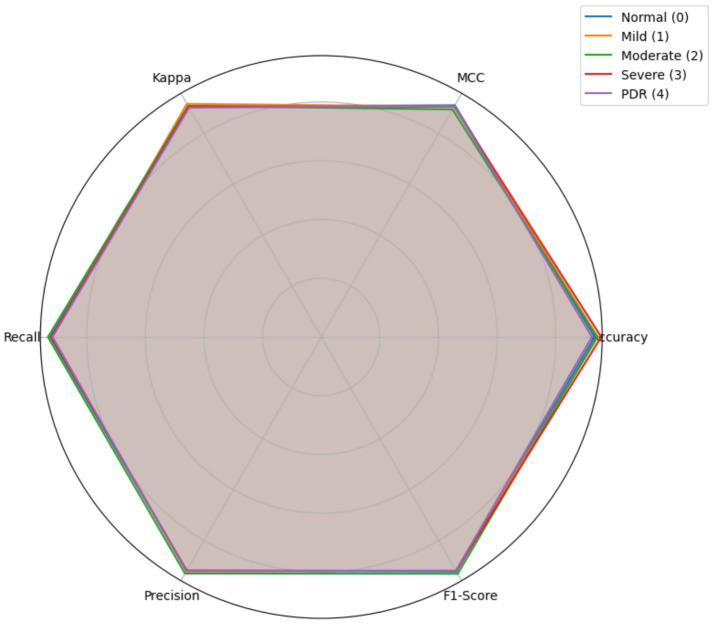
Findings of the multi-class performance evaluation – MESSIDOR-2 dataset.

Figure 7 offers the multi-class performance of the proposed DR severity grading model on the MESSIDOR-2 dataset. The symmetrical structure of the radar plot demonstrates the balanced and consistent performance across all classes without significant degradation in any metric. The high precision and F1-Score values confirm that the proposed DR severity grading model maintains a trade-off between sensitivity and specificity. Similarly, the high MCC and Kappa values validate the model’s robustness and agreement with ground truth labels, demonstrating the generalizability and stability of the proposed DR severity grading model.

Table 7 provides critical insights into the contribution of individual components in the proposed framework. Due to the limitations of traditional convolutional pipelines in modeling the complex hierarchical features, the baseline CNNs with dense layers achieved the lowest performance. The integration of ShuffleNet V2 with cKANs achieved a significant improvement by obtaining an accuracy of 90.18%, capturing discriminative local retinal features. The proposed DR severity grading model outperformed all other configurations due to the integration of ShuffleNet V2, LeViT, and cKANs models, yielding a stable and generalizable framework. To provide clearer insight into the computational differences between the two design choices illustrated in Figures 5a,b, we include a comparison of their parameter counts and FLOPs. A comparison of the architectures in Figures 5a,b highlights clear differences in their computational characteristics. The baseline CNN–ViT (ShuffleNet V2 + LeViT) model (Figure 5a) requires substantially more parameters and higher computational cost due to the fully connected transformations applied to high-dimensional feature vectors. This heavier architecture corresponds with lower performance, achieving only 91.59% accuracy and an MCC of 88.17%. In contrast, the proposed CNN–ViT configuration with cKANs (Figure 5b) replaces dense layers with spline-based univariate and multivariate compositions, which significantly reduces parameter count and overall FLOPs. Despite being computationally lighter, the cKAN-based design consistently outperforms the dense-layer baseline; the final IWOA-optimized model achieves 94.34% accuracy and an MCC of 90.53%. Intermediate variants using cKANs also surpass the dense baseline. These results demonstrate that cKANs provide both computational efficiency and superior representational power compared to traditional dense layers.

**Table 7:** Findings of the ablation study.

Classes	Accuracy (%) ± 95% CI half-width	MCC (%)	Kappa (%)	Recall (%)	Precision (%)	F1-Score (%)	SD
Proposed DR severity grading model	94.34 ± 1.03	90.53	90.55	93.02	92.47	92.74	0.010
Baseline CNNs + Dense Layer	87.20 ± 1.31	84.12	81.62	86.78	86.91	86.84	0.023
ShuffleNet V2 (No IWOA) + cKANs	90.18 ± 0.89	86.45	80.99	88.08	89.62	88.84	0.019
ShuffleNet V2 + LeViT (No IWOA) cKANs	91.59 ± 1.39	88.17	82.46	87.93	87.09	87.51	0.020
LeViT + cKANs	90.65 ± 1.84	86.32	83.89	89.23	90.54	89.88	0.029

Table 8 presents the performance of the proposed DR severity grading model with different hyperparameter strategies, indicating the significance of the proposed IWO strategy in delivering superior performance across all evaluation metrics. The IWO-optimized model achieves an exceptional accuracy of 94.34 ± 1.03 with the lowest SD of 0.010, reinforcing its robustness as an optimization mechanism. In contrast, the optimization methods, including Optuna, Hyperband, and BOHB, shows lower performance. Traditional population-based metaheuristics, such as Particle Swarm Optimization (PSO) and GA, yields the lowest value across all metrics, indicating their susceptibility to local optima and less effective parameter exploration. The improved exploration-exploitation of IWO enable the proposed DR severity grading model to identify the optimal hyperparameters of feature extraction pipeline, maximizing the classification performance of the cKANs model.

**Table 8:** Findings of the comparative analysis (proposed DR severity grading model with different hyperparameter optimization).

Classes	Accuracy (%) ± 95% CI half-width	MCC (%)	Kappa (%)	Recall (%)	Precision (%)	F1-Score (%)	SD
Proposed DR severity grading model with IWO fine-tuned feature extraction	94.34 ± 1.03	90.53	90.55	93.02	92.47	92.74	0.010
Proposed DR severity grading model with Optuna fine-tuned feature extraction	91.20 ± 1.25	86.10	85.70	89.20	88.90	89.00	0.018
Proposed DR severity grading model with Hyperband fine-tuned feature extraction	90.80 ± 1.30	85.70	85.30	88.70	88.40	88.50	0.019
Proposed DR severity grading model with BOHB fine-tuned feature extraction	91.50 ± 1.20	86.40	86.10	89.50	89.10	89.30	0.017
Proposed DR severity grading model with Whale optimization fine-tuned feature extraction	92.00 ± 1.15	87.10	86.80	90.00	89.70	89.80	0.015
Proposed DR severity grading model with PSO fine-tuned feature extraction	89.80 ± 1.40	84.20	83.90	87.50	87.10	87.30	0.021
Proposed DR severity grading model with GA fine-tuned feature extraction	90.10 ± 1.35	84.70	84.30	87.90	87.50	87.60	0.020

Table 9 highlights the performance of the proposed DR severity grading model against pre-trained deep learning models and recent DR detection approaches on the EyePACS dataset, highlighting the effectiveness of the proposed DR severity grading model in terms of accuracy, stability, and balanced performance. The conventional CNN architectures, including ResNet-50, DenseNet-161, and SqueezeNet exhibit limited performance, reflecting their inability in classifying the fundus images. Likewise, the recent DR approaches fail to match the stability and robustness of the proposed DR severity grading model, demonstrating the strengths of combined ShuffleNet V2, LeVit, and cKANs architectures.

**Table 9:** The comparative analysis outcomes using the EyePACS dataset.

Classes	Accuracy (%) ± 95% CI half-width	MCC (%)	Kappa (%)	Recall (%)	Precision (%)	F1-Score (%)	SD
Proposed Framework	94.34 ± 1.03	90.53	90.55	93.02	92.47	92.74	0.010
DenseNet-161	89.60 ± 1.35	83.90	83.50	87.20	88.10	87.65	0.022
Wahab Sait’s model (49)	89.10 ± 1.40	83.20	82.90	86.70	87.60	87.15	0.023
Ashwini and Dash’s model (54)	88.80 ± 1.45	82.80	82.40	86.20	87.10	86.65	0.024
Tian et al.’s model (26)	90.10 ± 1.30	84.40	84.10	88.10	88.90	88.50	0.021
EfficientNet Lite	90.80 ± 1.25	85.10	84.80	88.60	89.40	89.00	0.020
MobileNet V3	89.40 ± 1.40	83.40	83.10	87.10	88.00	87.55	0.023
SqueezeNet V1.1	87.90 ± 1.55	81.60	81.30	85.10	86.10	85.60	0.026
ResNet-50	90.30 ± 1.30	84.60	84.20	88.30	89.10	88.70	0.021
Gambhir et al.’s model (51)	88.70 ± 1.50	82.10	81.80	85.60	86.60	86.10	0.025
Nazih et al.’s model (22)	91.10 ± 1.20	86.00	85.70	89.10	89.90	89.50	0.019
Chintamreddy and Seshasayee ‘s model (27)	90.40 ± 1.25	85.00	84.70	88.40	89.20	88.80	0.020
Kakade and Kakade’s model (55)	89.50 ± 1.35	83.80	83.50	87.40	88.20	87.80	0.022
Ikram and Imran’s model (56)	90.70 ± 1.25	85.30	85.00	88.60	89.40	89.00	0.020
Zhang et al.’s model (48)	90.90 ± 1.20	85.60	85.30	88.80	89.60	89.20	0.019

**Figure 8:**
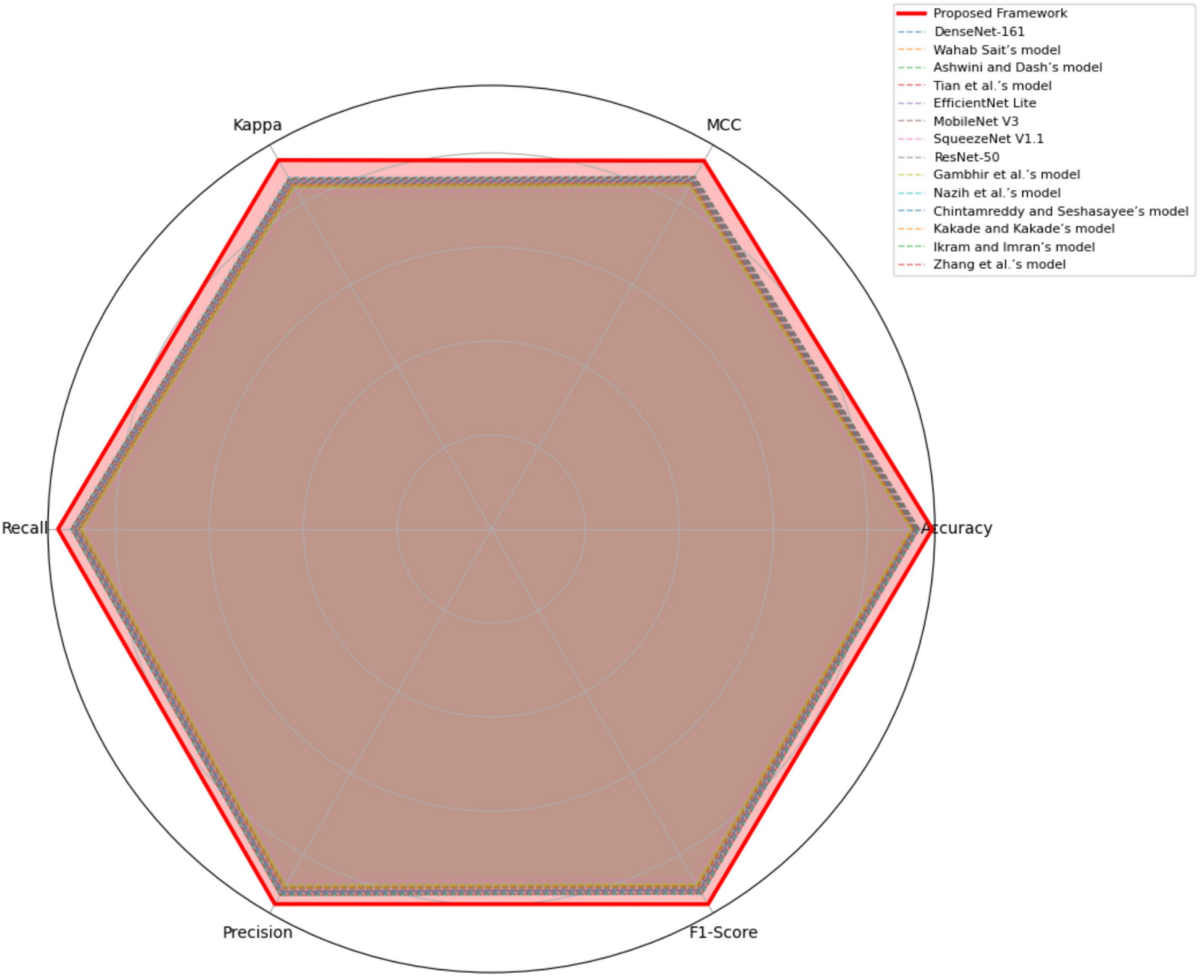
The comparative analysis outcomes using the MESSIDOR-2 dataset.

Figure 8 visualizes the comparative analysis of the proposed DR severity grading model against baseline models using the MESSIDOR-2 dataset, providing a holistic comparison of model robustness and generalization. The proposed DR severity grading model outperforms the baseline models across the evaluation metrics by achieving an accuracy of 93.84 ± 1.03, affirming its strength and adaptability to external validation dataset. Baseline CNN architectures report lower recall and precision values. Similarly, the lightweight models were unable to achieve the balance between sensitivity and specificity. The reliance on the single ViTs or CNN architectures reduce the performance of the existing approaches.

**Figure 9:**
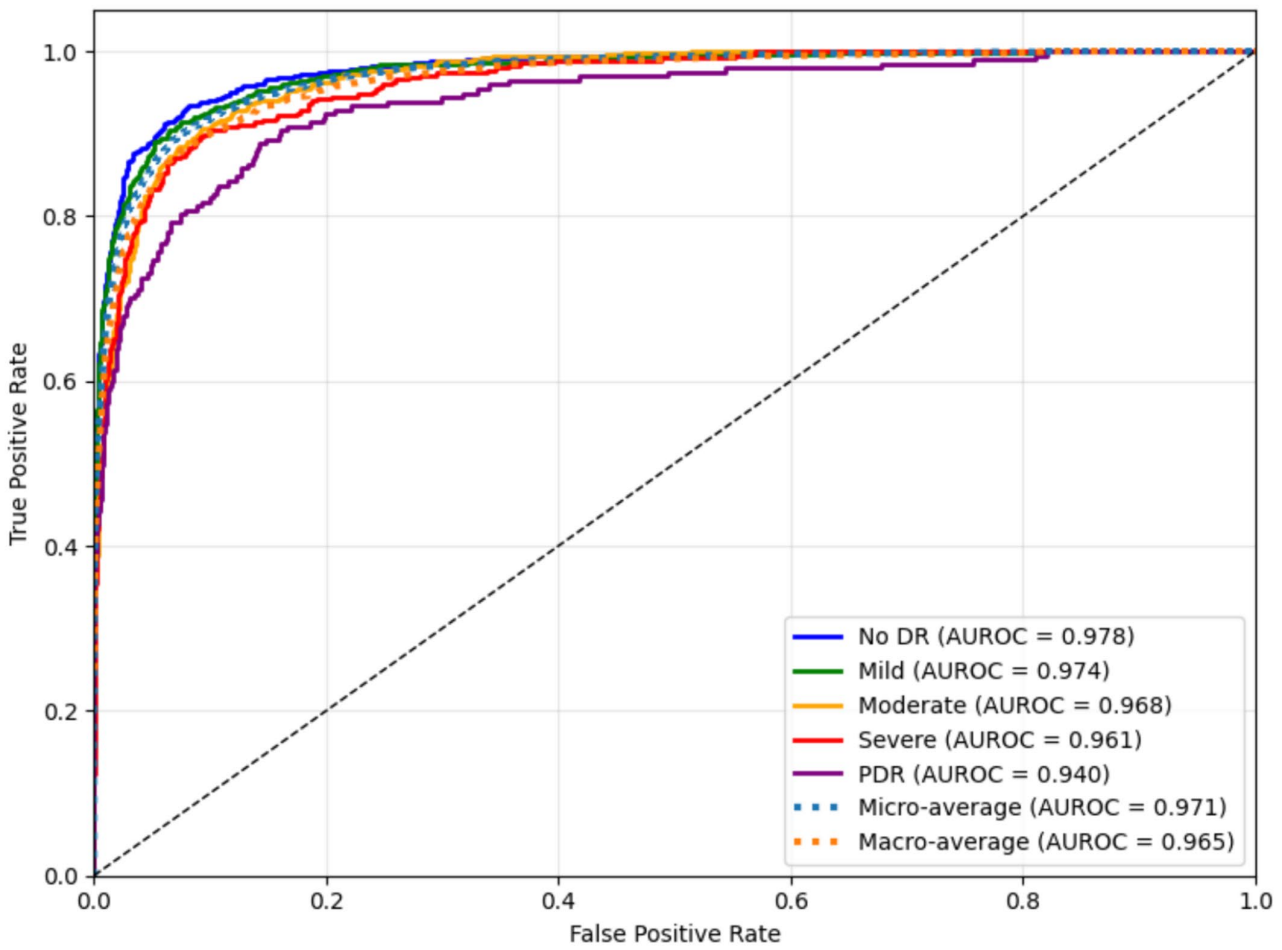
AUROC values for multi-class DR classification using the proposed framework.

Figure 9 illustrates the AUROC values for the five DR severity levels, confirming the strong discriminative ability of the model across all categories. The model achieved the highest AUROC (0.978) for the No DR class. The findings indicate that the model is highly reliable in differentiating healthy retinal and pathological images. The consistent high AUROC values highlight the model’s sensitivity to early disease features, which is essential for timely clinical intervention. The slightly lower AUROC for PDR reflects the inherent data imbalance, affirming robust generalization.

**Figure 10:**
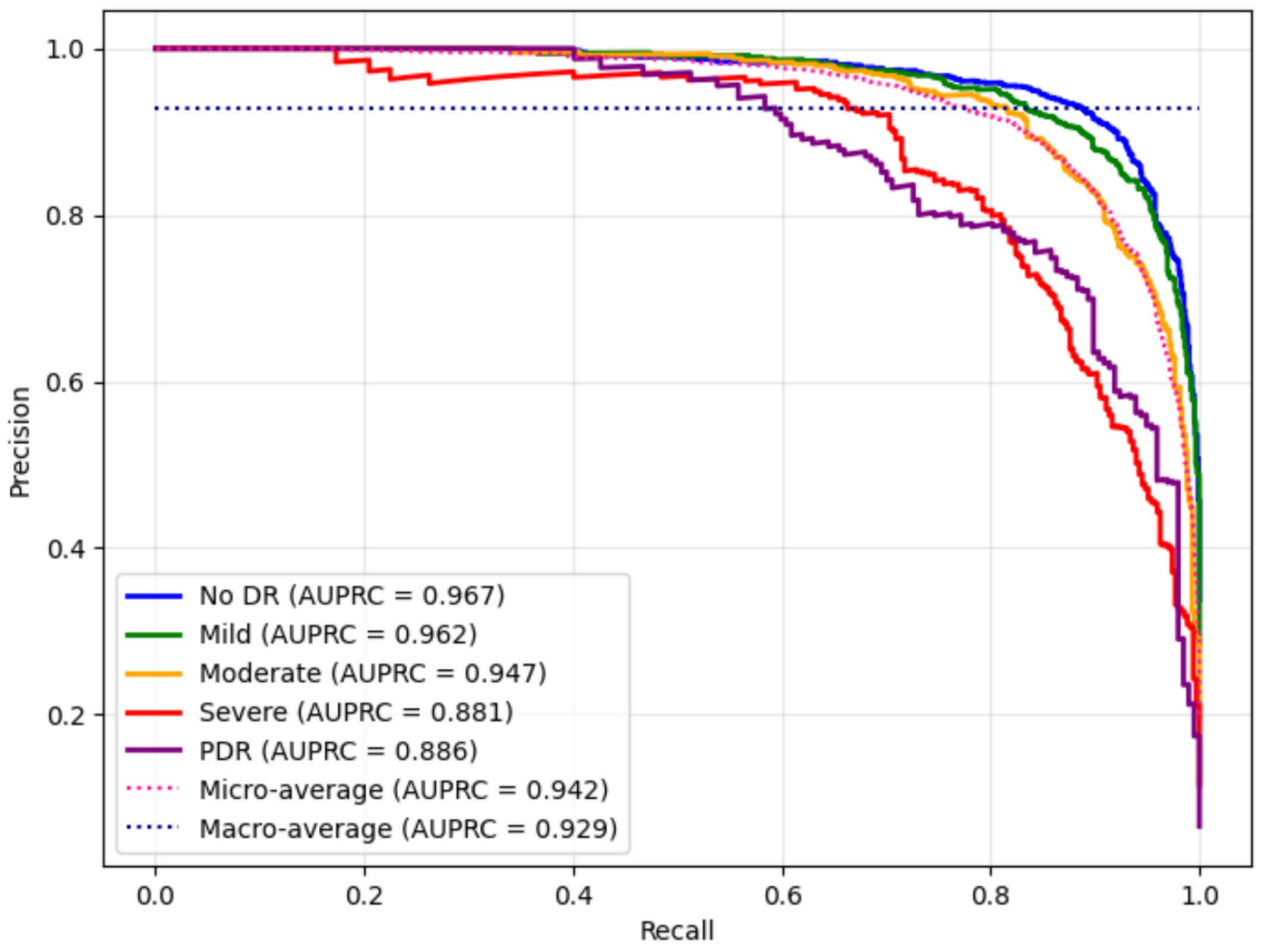
AUPRC values for multi-class DR classification using the proposed framework.

Figure 10 presents the AUPRC values for the proposed DR severity grading model, emphasizing the trade-off between recall and precision. The highest AUPRC values of the No DR and Mild DR classes confirm the model’s reliability in distinguishing normal retinal images from pathological cases. Due to fewer severe and PDR samples, the model achieved slightly lower AUPRC values for severe and PDR classes.

Table 10 highlights the computational efficiency of the DR severity grading approaches, emphasizing on model size, computational cost, and inference speed. The GFLOPs count in Table 10 represents the computational cost of the inference-time architecture only without the one-time overhead of the off-line optimization process provided by the IWO algorithm. Since IWO is only performed during the training phase to find an optimal set of hyperparameters of the backbone, it is not a part of the computation burden at the deployment phase. Therefore, the GFLOPs in Table 10 represent the final tuned model executed during the inference phase and do not include the exploration cost of fitness evaluations performed during the IWO iterations. This distinction guarantees that the reported complexity is directly comparable with state-of-the-art methods, which also omit hyperparameter tuning cost in GFLOPs calculations. The proposed DR severity grading model maintains a balance between performance and efficiency, ensuring scalability for deployment in real-time DR screening environments. DenseNet-161 and EfficientNet-B7 demand higher computational resources with slower inference speeds. SqueezeNet V1.1 and MobileNet V3 require fewer parameters. However, the limited feature representational power affects their generalization performance. Nazih et al. (22) and Zhang et al. (48) models present the importance of transformer architectures. However, these models demand considerable computational overhead. In contrast, the proposed DR severity grading model delivers exceptional outcomes with limited computational resources, underlining its novelty and practicality.

**Table 10:** Computing power of DR severity detection frameworks.

Models	Parameters [in Millions (M)]	GFlops [in Giga (G)]	GPU speed (Images / Sec)
Proposed Framework	21 M	4.8G	91
DenseNet-161	31 M	40.8G	72
Wahab Sait’s model (49)	77 M	193G	54
Ashwini and Dash’s model (54)	45 M	105G	61
Tian et al.’s model (26)	27 M	89G	69
EfficientNet B7	66 M	193G	55
MobileNet V3	5.4 M	1.15G	76
SqueezeNet V1.1	1.2 M	4.3G	80
ResNet-50	25.6 M	21.4G	70
Gambhir et al.’s model (51)	52 M	110G	66
Nazih et al.’s model (22)	33 M	98G	60
Chintamreddy and Seshasayee ‘s model (27)	38 M	115G	62
Kakade and Kakade’s model (55)	41 M	120G	63
Ikram and Imran’s model (56)	35 M	102G	65
Zhang et al.’s model (48)	37 M	108G	64

The interpretability and reliability of the proposed DR severity grading model is highlighted in Table 11. Through the visualization of regions associated with the model’s decision, the proposed DR severity grading model provides accurate predictions and transparent reasoning aligning with clinical knowledge. For instance, the absence of highlighted pathological regions for No DR cases indicate that the model correctly focuses on the optic disc and macula. The SHAP overlays assist clinicians with visual justification, improving explainability of the proposed DR severity grading model. The progression changes in overlays demonstrate the key role of the proposed DR severity grading model in stratifying patients by risk, allowing healthcare centers to prioritize resources effectively.

**Table 11:** Sample inputs and outputs with SHAP overlaps.

Ground truth	Predicted image	Model’s prediction	Explanation
No DR	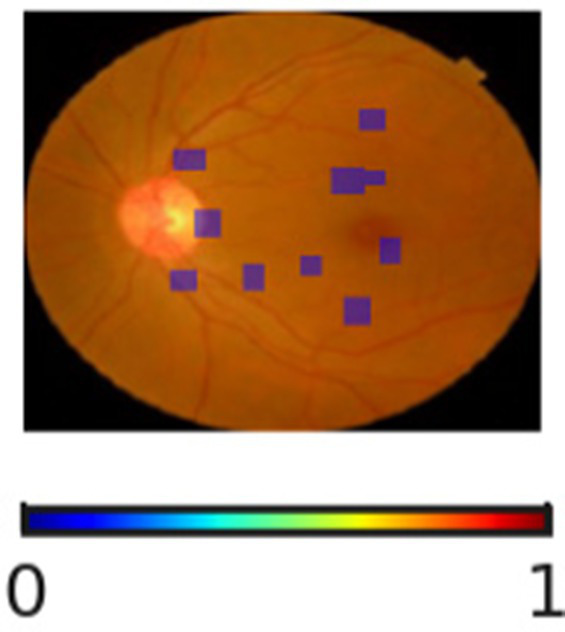	No DR	The SHAP overlay indicates the absence of microaneurysms, hemorrhages, or exudates. The proposed DR severity grading model focusses on the optic disc and macula without evidence of pathological lesions.
Mild DR	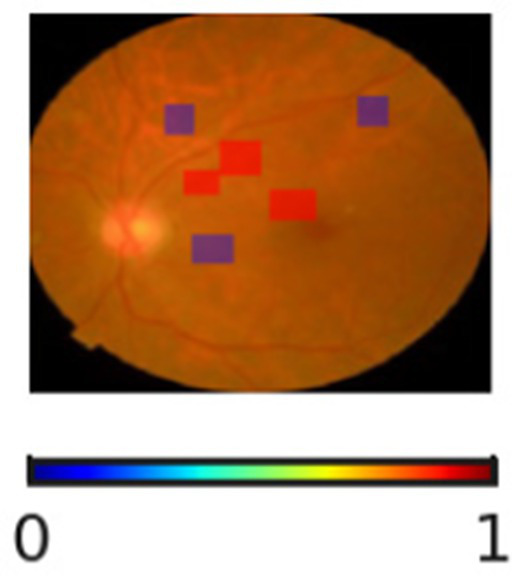	Mild DR	The outcome highlights small and scattered regions corresponding to initial microaneurysms, justifying the model’s decision.
Moderate DR	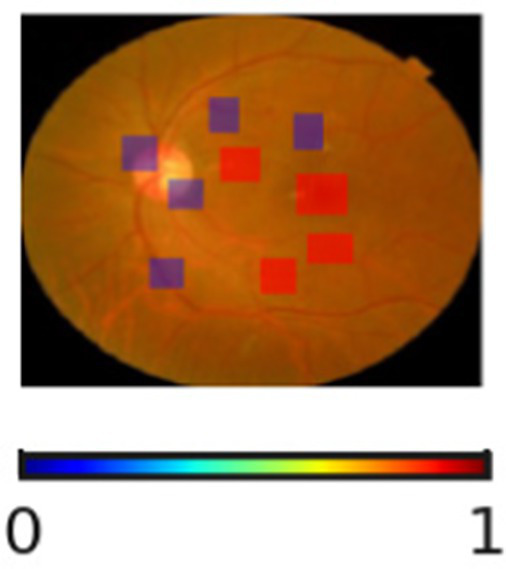	Moderate DR	The predicted image emphasizes multiple retinal regions that indicates denser clusters, reflecting increased lesion and validating the moderate DR classification.
Severe DR	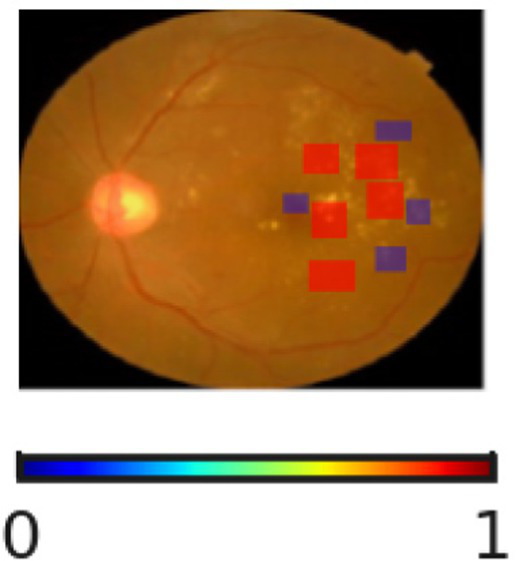	Severe DR	The outcome shows extensive highlighted regions near vascular arcades corresponding to widespread hemorrhages and exudates.
Proliferative DR	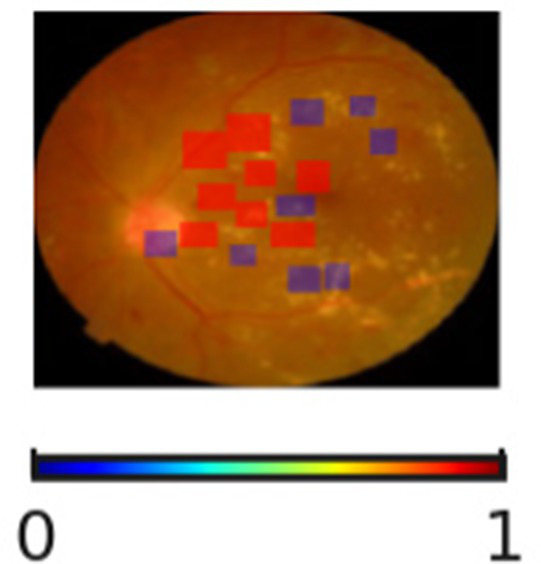	Proliferative DR	The SHAP overlay strongly reflects neovascular regions and widespread lesions, capturing advanced pathological variations.

## 5 Discussion

A DL-based model for classifying the severity grades of DR using the fundus images was introduced in this study. There were four stages to the suggested architecture: preprocessing images, extraction features, DR detection, and performance evaluation. The images were acquired from the EyePACS dataset. The CLAHE technique was used to enhance the quality of the fundus images. The enhanced Whale optimization method was used to determine the crucial characteristics of the fundus images. CKAN was used to find the severity levels. The recommended image preprocessing has enhanced the image quality in order to improve the proposed DR severity grading model’s performance. We implemented the Cauchy mutation to improve the update location capability of the conventional Whale optimization algorithm. The IWO algorithm efficiently balances the tradeoff between exploration and exploitation, prioritizing the key hyperparameters that substantially influence the performance of the feature extraction process. Using the hybrid ShuffleNet V2-LeViT’s feature extraction approach, a specific collection of crucial features were identified, assisting the suggested model in delivering insights with limited processing resources. Furthermore, the BOHB algorithm was used to optimize the parameters of the cKANs model. The experimental results underscores the importance of the proposed approach in identifying DR through the analysis of fundus images. The study’s findings suggest that the framework can accurately determine the severity grade of diabetic retinopathy.

Li et al. (19) employed an ensemble approach of five classification models. In addition, they used the Inception V4 model to boost the model’s performance. A primary dataset of 8,293 retinal fundus images and MESSIDOR-2 datasets were utilized to evaluate the performance of the DR severity grade prediction model. The model achieved an AU-ROC value of 97.7% with CI (94.3–95.1) on the MESSIDOR-2 dataset. In addition, it obtained a sensitivity value of 92.2%. However, the ensemble learning approach reduced the model’s performance. Inception V4 required a significant number of parameters. The network size demanded massive computational resources compared to the proposed DR severity grading model. Ramos et al. (20) built a DR detection model using the pre-trained CNN models. They employed ResNet-50 and DenseNet 161 to classify the images. The detection model was generalized using EyePACS (31) and MESSIDOR-2 datasets. The fine-tuned ResNet-50 model obtained an AU-ROC value of 85%, and DenseNet-161 achieved an AU-ROC value of 89% on MESSIDOR-2. However, ResNet-50 and DenseNet-161 models demand a larger dataset and huge computation costs for generating the outcome. The training process for ResNet-50 and DenseNet 161 was time-consuming and complex. The integration of these models affected the prediction accuracy. On the other hand, the proposed framework produced a superior result on the MESSIDOR-2 dataset.

Islam et al. (21) introduced a DL-based DR detection framework. They used the Xception CNN model to construct the framework. In addition, supervised contrastive learning was utilized to handle the noisy data and hyper-parameter variations. The model was evaluated using the APTOS-2019 and MESSIDOR-2 datasets, respectively. It obtained an overall accuracy of 84.36% and an AU-ROC value of 93.82% on the APTOS -2019 dataset. On the MESSIDOR-2 Dataset, this model has obtained an overall accuracy of 74.21% and AU-ROC of 87.26%. In contrast, the proposed DR severity grading model produced a better outcome by achieving an average accuracy of 93.84% on the MESSIDOR-2 dataset. The contrastive learning algorithm was designed for the specific datasets. It required substantial computational power and memory. Thus, the model produced low prediction accuracy compared to the proposed DR severity grading model. The suggested image quality enhancement assisted the proposed framework in overcoming the limitations of the existing methods.

Kassani et al. (14) proposed an extraction technique for extracting the image features. They modified the Xception architecture and integrated it with the multi-layer perceptron to classify the DR severity grade. They followed the hyper-tuning technique to improve the Xception network’s ability to classify the images. The model obtained an average classification accuracy of 83.09%, a sensitivity of 88.24%, and a specificity of 87.00%. Multi-layer perceptron-based model is more challenging to implement in a resource-constrained environment.

Chaturvedi et al. (18) employed DenseNet 121 to develop the DR detection framework for binary and multi-class classification using the APTOS-2019 dataset. They generalized the model on the APTOS-2019 dataset using 15 epochs with a kernel size of 3 × 3. The DenseNet layers processed and forwarded the images to the subsequent layers. They used the dropout and global average pooling 2D layers to address the overfitting issues. The model obtained a considerable performance. However, DenseNet 121 has become memory intensive due to the dense connections and integration of feature maps. Alyoubi et al. (17) developed a CNN15-based DR detection model for grading the DR severity. They followed the feature extraction method to generate the feature maps. They combined CNN 512 and the Yolo V2 models for the image classification. In order to customize Yolo V2 for DR severity classification, the researchers conducted extensive image preprocessing and architectural modification by integrating CNN 512 mode. The extracted features may not be practical for image classification, which may lead to a high value of false positives. The model obtained an average accuracy of 84.1% and a specificity of 97.3%. In contrast, the suggested framework applied the feature selection technique to extract the feature maps. Thus, the proposed framework obtained a superior accuracy compared to the existing models.

Panwar et al. (11) employed the InceptionNet V3 model for classifying the DR severity level. They compared their model with the AlexNet and VGG16 based image classification models. The performance of the InceptionNet V3 Networks outperformed the AlexNet and VGG16 models. Mobeen-Ur-Rehman et al. (2019) (34) built a DR detection framework using a five-layered CNN model with two convolutions and three fully connected layers. The customized CNN produced significant results compared to the AlexNet and VGG16 models. Rahim et al. (35) applied data augmentation and image preprocessing techniques to improve the classification accuracy of the CNN model. They generalized their model on the private dataset and achieved an accuracy of 77%. The integration of additional layers has diminished the prediction accuracy of the ResNet model. In addition, AlexNet required a larger number of parameters, leading to the memorization of training samples. The shortcomings of the ResNet and AlexNet models have reduced their overall performance. To overcome this limitation, we applied early stopping and quantization-aware training strategies in the proposed DR severity grading model.

Wahab Sait (49) developed a lightweight model for DR severity using the MobileNet backbone. Compared to the proposed DR severity grading model, Wahab Sait’ model achieves a lower accuracy due to its limited feature extraction capability. The lack of an early stopping strategy has increased the training time to learn the intricate features. Tian et al. (26) and Huang et al. (50) proposed fine-tuned CNN models for detecting DR severity. However, the limited training samples have reduced the generalizability of these models. In addition, the fine-tuned models failed to identify optimal hyperparameter settings due to insufficient exploration of parameters compared to the proposed DR severity grading model. Gambhir et al. (51) employed the ShuffleNet V2 model to extract critical features. The sensitivity to hyperparameters has reduced the model’s performance. The depthwise separable convolutions and channel shuffling have limited its flexibility in learning feature representation compared to the proposed DR severity grading model. The proposed framework obtained a superior outcome on the MESSIDOR-2 and EyePACS datasets compared to the recent models. In addition, the proposed framework allows the developer to build a mobile application to monitor the patients. The findings of the comparative analysis indicates the potential of the streamlined proposed framework in detecting DR severity grades. The lightweight ShuffleNet V2 demands limited parameters and FLOPS to classify the images. Using the proposed framework, a cost-effective DR screening application can be developed. It can support developing countries in providing effective treatment for individuals. Moreover, the mobile-based screening system can assist healthcare practitioners in reaching patients in remote areas. The proposed DR detection framework can be implemented in real-time to diagnose DR patients.

The proposed DR severity grading model has some limitations. The datasets were highly imbalanced. In order to overcome the limitations, we employed image augmentation techniques. The images were captured using various types of devices. Thus, the images were varied in size and resolution. Image preprocessing and normalization techniques were required to improve image clarity. An extended image preprocessing is required for generalization in new datasets. We encountered challenges in converting features into one-dimensional vectors using the flattened layer during the feature extraction. However, integrating batch normalization and dropout layers minimized the computational complexities of the conversion process. The proposed DR severity grading model may demand a dedicated image preprocessing in order to maintain optimal performance. The fundus images may contain tiny or complex patterns that mimic DR severity patterns, resulting in false positives. The model was generalized in the EyePACS dataset. However, generalizing the model in diverse datasets is essential for implementing it in real-time settings. An additional computing resource is necessary to streamline the proposed framework in processing the fundus images. The lightweight ShuffleNet V2 requires an external CNN model for the larger datasets. We used a quantization-aware training approach to reduce the computational complexities of the proposed DR severity grading model. Integrating the proposed DR severity grading model for DR severity analysis and individualized treatment approaches can be difficult due to infrastructure, training, and data protection issues.

Integrating multi-modal data sources, including optical coherence tomography and electronic health records may capture diverse biomarkers associated with DR severities. By leveraging federated learning setting, the generalizability of the proposed DR severity grading model can be improved while preserving patient privacy. The enhancement of explainability layer through the incorporation of layer-wise relevance propagation with SHAP overlays can provide greater clinical transparency. Exploring adaptive hybrid metaheuristics can dynamically balance exploration and exploitation, leading to convergence stability and accuracy.

## 6 Conclusion

In this study, a novel multi-class DR severity grading model integrating hybrid ShuffleNet V2-LeViT-feature extraction, optimized using an IWO algorithm and cKANs classifier head, is introduced. Using a five-fold cross validation strategy, the model was trained and tested using the EyePACS dataset. In addition, it was generalized on the MESSIDOR-2 dataset, achieving a generalization accuracy of 93.84% with limited computational overhead. The SHAP overlay layer offered insights into the model’s decision-making process, fostering trust and clinical applicability. Unlike the existing approaches, the proposed DR severity grading model used local spatial features and global contextual dependencies, enhancing the cKANs classification performance. The application of the cKANs classifier have addressed the limitations of gradient boosting and dense layers in handling complex and non-linear decision boundaries. The experimental findings emphasize the model’s strength in detecting subtle early lesions, contributing to early DR detection, risk stratification, and disease progression monitoring. However, the model performance in real-world settings may be influenced by factors such as class imbalance, device-specific artifacts, and variations in imaging conditions. Integrated multi-modality, real-time deployment on devices with limited resources, and validation through large-scale clinical trials should be the primary focuses of further investigations. The proposed study sets a platform for trustworthy, interpretable, and generalizable AI solutions in ophthalmology, reducing preventable blindness across the globe.

## Data Availability

The original contributions presented in the study are included in the article/supplementary material, further inquiries can be directed to the corresponding author.
